# Addressing global challenges: How does the integration of rural industries in China enhance agricultural resilience?

**DOI:** 10.1371/journal.pone.0327796

**Published:** 2025-07-07

**Authors:** Xiaoli Zhou, Mingyang Han

**Affiliations:** 1 College of Management, Minzu University of China, Beijing, China; Peking University, CHINA

## Abstract

Promoting the integrated development of rural industries represents a crucial pathway for accelerating the modernization of the entire industrial chain and consolidating and enhancing agricultural resilience. This study constructs provincial-level panel data for China spanning 2012–2022 and employs a multidimensional, visualization-based, and spatial research paradigm to comprehensively examine the impact effects and mechanisms through which rural industrial integration empowers agricultural resilience in China. The findings reveal that, first, rural industrial integration can effectively enhance agricultural resilience levels, with stable economic returns and diversified functional development serving as key pathways for improving agricultural resilience. Second, the eastern and western regions have unleashed agricultural resilience potential through superior policy and environmental advantages, while the relatively homogeneous grain structure and path dependence in the central and northeastern regions have prevented agricultural industrial integration from effectively achieving expected outcomes. Third, structural rationalization has improved resource allocation efficiency, but industrial structure advancement and industrial structure sophistication have actually diminished the enhancement effects on agricultural resilience. Fourth, urbanization has led to adverse resource competition, reducing the positive impact of rural industrial integration on agricultural resilience levels. Fifth, the enhancement of agricultural resilience through rural industrial integration demonstrates geographically-distant resource spillover effects to neighboring regions. This research provides an important theoretical framework and practical paradigm for global agricultural transformation, offering particularly significant strategic guidance for developing countries in building resilient agricultural systems, addressing climate change impacts, and ensuring food security.

## 1. Introduction

Confronted with multiple challenges including global climate change, resource and environmental pressures, international trade frictions, and geopolitical complexities, agricultural modernization faces increasingly volatile and uncertain factors [1–3]. To date, climate change has caused global production of major crops such as rice and wheat to decline by approximately 0.3% and 0.9%, respectively. Multiple model predictions indicate that by the end of the 21st century, global production of staple crops including rice, wheat, and maize is expected to decline, with yield reductions ranging from 7% to 25% and overall agricultural productivity decreasing by 2% to 15% [4,5]. In contrast to the global declining trend in rice and wheat production, China’s total grain output reached 706.5 billion kg in 2024, representing an increase of 11.09 billion kg from the previous year, a growth rate of 1.6%, marking the first time crossing the 700 billion kg threshold and demonstrating “Chinese resilience” [6].

China’s agricultural civilization has accumulated thousands of years of heritage, but due to its relatively late start in modernization, it frequently encounters multiple uncertainty shocks [7]. Since the release of the Central Government’s “No. 1 Document” in 2015, the Chinese government has vigorously promoted the integrated development of primary, secondary, and tertiary industries in rural areas (referred to as “rural industrial integration”), which has become a critical node for establishing a modernized food security system with distinctive characteristics and ensuring its dynamic sustainable development. This is primarily achieved through promoting the integrated development of rural agriculture, processing industries, and service industries, thereby enhancing the stability and diversity of rural industrial chains as well as the security of supply chains [8,9]. Currently, China’s agricultural modernization process continues to accelerate, having preliminarily established a diversified modern industrial system centered on industrialized operations, specialized production, and multifunctional expansion [10]. However, the vulnerabilities in key links such as production, distribution, and consumption have not yet achieved fundamental improvement [11–13]. Therefore, rural industries have become an important driving force for high-quality agricultural development through mutual penetration and cross-integration. Therefore, rural industries have become an important driving force for high-quality agricultural development through mutual penetration and cross-integration. It is foreseeable that rural industrial integration will become a new core engine for building agricultural resilience, providing more solid assurance for addressing future complex and dynamic challenges.

Currently, research on agricultural resilience in China fails to clearly reflect the pathway analysis and practical significance of rural industrial integration development. Huang [14] observed that despite China being an agricultural powerhouse, China’s agricultural labor productivity is less than one-third that of manufacturing and service industries, representing only 25.3% of non-agricultural industries, while developed countries (such as the United States and Germany) maintain agricultural labor productivity at approximately 65% or more relative to non-agricultural industries, revealing substantial disparities in industrial resource allocation and development. Research on these two aspects primarily focuses on the analysis of three characteristics: “industrial chain optimization—functional expansion—benefit mechanisms,” neglecting the diversified revenue effects brought by industrial integration in rural areas, with research paradigms and frameworks being relatively singular. Furthermore, unlike Yang [15] who approached the issue solely from an agricultural economic perspective, this study draws on Meuwissen [16] to construct a comprehensive agricultural resilience assessment framework that encompasses market volatility, policy environment changes, labor shortages, and other issues. Moreover, differing from traditional perspectives such as industrial structure [17], industrial agglomeration [18], or industrial innovation [19], this paper incorporates multi-dimensional industrial integration into the examination scope of agricultural resilience enhancement. This study primarily attempts to answer the following questions: Do rural industrial integration and its multi-dimensional heterogeneity effectively drive the improvement of agricultural resilience levels? How does industrial structure dynamically adjust during the integration process to be effectively embedded in the pathway between rural industrial integration and agricultural resilience enhancement? Additionally, do rural industrial integration and agricultural resilience exhibit potential spillover effects? Do they generate certain radiating and driving effects on the development of neighboring regions? In brief, this article conducts a comprehensive assessment of agricultural resilience from the perspective of industrial integration, aiming to explore whether rural industrial integration can become a core pillar of agricultural resilience and provide scientific evidence and practical guidance for the formulation and implementation of policies related to enhancing global agricultural development resilience and agricultural food system transformation.

## 2. Literature review

The concept of “resilience” originated in the field of physics and was later introduced to ecology by Holling [20], who focused on analyzing ecosystems’ capacity to maintain, recover, and innovate following natural and anthropogenic shocks. Subsequently, this concept has gradually extended to fields such as economics and sociology [21,22].

During the early development of resilience theory, Darnhofer [23] theoretically constructed a framework for assessing farm sustainability, emphasizing the importance of diversity and redundancy in agricultural resilience. However, similar to Torrico [24], this research lacked precise quantitative indicators, limiting its widespread application across different agricultural contexts. Recently, Meuwissen [16] developed a framework for assessing European agricultural system resilience from multiple levels of agricultural systems (such as farms, farmers, and supply chains), providing a more standardized definition of agricultural resilience. Subsequently, Zampieri [25] proposed an easily calculable index to assess crop production resilience (the reciprocal of the squared coefficient of variation) and quantified the added value of crop diversity. In recent years, numerous scholars have focused on the influencing factors of economic and industrial resilience when measuring agricultural resilience, considering its characteristics of vulnerability, cyclicality, and complexity [26,27]. Research demonstrates that agricultural economic and industrial resilience are not only driven by internal production characteristics but are also significantly constrained by external production environments. Research demonstrates that agricultural economic and industrial resilience are not only driven by internal production characteristics but are also significantly constrained by external production environments. The external production environment primarily encompasses support from elements such as policy support, social capital, climate and ecological environment, financial support, and infrastructure [28–31]. It is evident that traditional agricultural resilience research has primarily focused on systems’ internal capacity for self-recovery, adaptation, and shock response, with emphasis on coping strategies for climate change, ecological risks, and economic crises, concentrating on systemic resilience factors such as diversity, redundancy, and robustness.

**Fig 1:**
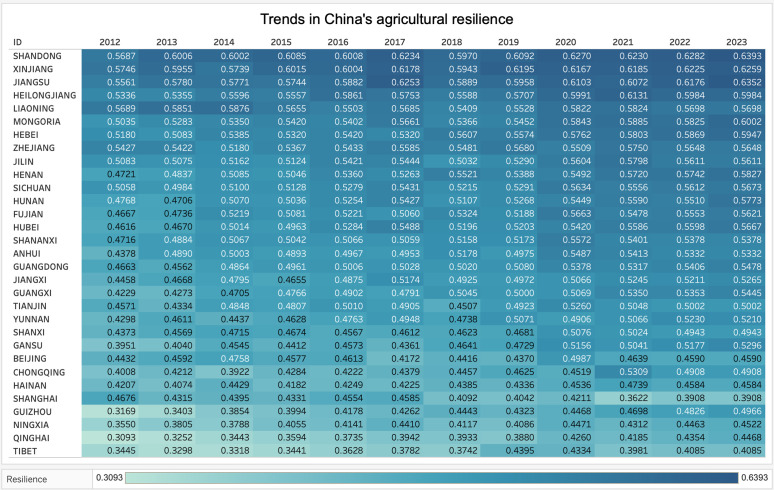
Trends in agricultural resilience development.

With the deepening of global economic integration, an increasing number of scholars have begun to focus on enhancing agricultural systems’ capacity for risk resistance, recovery, and innovation through cross-industry collaboration [22]. Rural industrial integration represents an important exploration in constructing modern agricultural industrial systems and accelerating the transformation of agricultural development modes [32]. Research on rural industrial integration can be traced back to the concept of “six-sector industry” proposed by Japanese scholar Imamura Naraomi, which is considered an important starting point for rural industrial integration research [33]. This process involves not only the integration of traditional agriculture with emerging industries but also the synergistic development of various links within agriculture itself. For example, Fitz-Koch [34] demonstrated that vertical integration, value chain expansion, and market-oriented development improvements can significantly enhance agricultural industrial resilience and create sustainable returns. Subsequently, Chinese scholar Huo [35] proposed a comprehensive collaboration model centered on farmer cooperatives, establishing organizational guarantees for agricultural resilience through systematic strategies including implementing strict regulatory systems, developing diversified additional revenue channels, and optimizing industrial synergy coefficients. Building upon this foundation, Donner [36] advanced theory to the practical level by proposing six different business models including biogas plants, upcycling entrepreneurship, and agricultural cooperatives, demonstrating how to create new agricultural business formats through circular economy and biomass value addition. It is evident that current rural industrial integration development emphasizes introducing modern industrial organizational approaches such as industrial chains and value chains into agriculture, achieving significant improvements in agricultural resilience levels through pathway optimization including chain extension, stakeholder participation, business format development, model innovation, and factor activation to optimize resource utilization and enhance market adaptability (Fig 1).

Research on rural industrial integration and agricultural resilience primarily focuses on analyzing three key characteristics: “industrial chain optimization—functional expansion—benefit mechanisms.” For example, Šūmane [37] argued that diversified knowledge integration, indigenous knowledge, and multi-stakeholder collaborative knowledge network adaptation help enhance agricultural systems’ resilience and adaptability under conditions of complexity and uncertainty.However, Chinese scholars typically emphasize localized research orientations, focusing on exploring local institutional models and their evolutionary mechanisms. For instance, Ren [38] found that contract farming models significantly improved farmers’ green productivity, contributing to cost reduction and efficiency enhancement, green production, and ultimately promoting food security and sustainable agricultural development. In fact, rural industrial integration, as a complex socioeconomic phenomenon, often transcends administrative boundaries when influencing individual entities, demonstrating significant spillover effects across geographic spaces [39]. Research demonstrates that the spatial spillover effects of rural industrial integration are primarily achieved through four mechanisms: knowledge sharing, technology diffusion, market linkage, and factor mobility [40–42]. Further evidence confirms that regions with higher levels of industrial integration can drive surrounding areas to jointly improve agricultural resilience levels through industrial chain extension and cross-regional cooperation, creating favorable patterns of regional coordinated development.Moreover, industrial integration in China’s eastern coastal regions demonstrates more significant radiating and driving effects on surrounding areas [43,44], while central and western regions exhibit patterns that combine endogenous development with external absorption.

With the deepening of resilience research, simple resistance to external shocks can no longer meet the long-term development needs of agricultural systems, making prevention and transformation of rural industrial environments equally important. The potential contributions of this paper are primarily threefold: First, utilizing Chinese provincial panel data from 2012 to 2022, it empirically examines the direct effects of rural industrial integration development on agricultural resilience while considering heterogeneity across diversified levels, domains, and regions. Second, it employs “industrial structure transformation” to further validate the intrinsic transmission mechanism of rural industrial integration development → industrial structure transformation → agricultural resilience. Additionally, the research examines “urbanization level” (calculated as permanent residents minus registered population divided by permanent residents) as an external moderating effect, attempting to reveal the dynamic relationship of urbanization level in the process of rural industrial integration enhancing agricultural resilience. Third, considering the spatial correlation characteristics of regional economic activities, the paper attempts to construct a spatial spillover effect model of rural industrial integration and agricultural resilience using “spatial economic distance,” providing data support for achieving regional coordinated development.

## 3. Theoretical analysis and hypotheses

**Fig 2:**
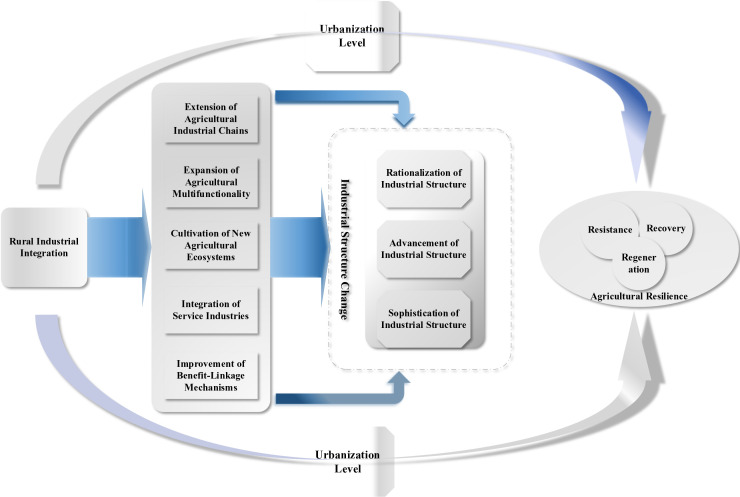
Theoretical framework diagram.

Rural industrial integration encompasses not only vertical extension across various links in industrial chains but also deeper horizontal expansion [45]. This integration includes vertical integration formed around key stages of agricultural products—production, processing, storage and transportation, and sales—gradually expanding and extending the agricultural value chain to achieve seamless connectivity from “field to market” [43]. Furthermore, through developing emerging business formats such as precision agriculture, agricultural tourism, and ecological agriculture, rural areas are no longer merely bases for grain production but are gradually becoming horizontal integration platforms that combine ecological tourism and cultural experiences [35]. The current penetration of new technologies such as big data and artificial intelligence has significantly improved production efficiency across agricultural sectors, achieving a leap from traditional agriculture to smart agriculture [38]. As challenges such as climate change, natural disasters, and resource scarcity intensify, rural industrial integration as a new development model has gradually become an important mechanism for agriculture to address multiple risk shocks (Fig 2). Research demonstrates that the diversified support systems, collaborative mechanisms, and stable industrial chains constructed through rural industrial integration can effectively reduce the impact of external shocks and maintain normal system operations [22]. Resource allocation and collaborative mechanisms can effectively address production disruption issues commonly encountered after disasters, enabling agricultural systems to recover rapidly post-disaster and avoid prolonged stagnation. The multi-dimensional innovation models and multifunctional development of rural industrial integration bring new growth drivers to agricultural systems, not only enhancing agricultural system adaptability but also promoting sustainable development directions. It is evident that rural industrial integration, as a complex modern integrated development model, can comprehensively enhance agricultural resistance, recovery, and regenerative capacity. Based on this analysis, the following hypothesis is proposed:

H1: Rural industrial integration and its multi-dimensional heterogeneity significantly enhance agricultural resilience.

Since China’s reform and opening up, the country’s economic structure has undergone significant changes, with one prominent feature being industrial structure transformation, specifically manifested through large-scale labor migration from agricultural to non-agricultural sectors [46]. Research demonstrates that industrial structure transformation contributes to improving rational resource allocation efficiency, extending industrial value chains, and advancing industrial modernization upgrades, providing multi-dimensional support for the risk resistance and adaptive capacity of traditional agriculture, industry, and service sectors [47–49]. Subsequently, with the continued deepening of economic development models across East Asian countries (such as Japan), their industrial development processes have evolved toward rationalization, sophistication, and advancement [50]. For example, rational industrial structure can achieve optimal allocation among agriculture, industry, and service sectors, thereby enhancing the risk resistance capacity of the entire economic system [51]. However, Navas [52] argued that extending industrial chains and increasing product added value enables rural industries to gradually enter high-value-added processing and sales segments, thereby gaining stronger competitiveness in unstable market environments. Furthermore, Li [53] emphasized that achieving refined management and intelligent production through digital tools and information technology can improve industrial environmental adaptability. It is evident that industrial structure transformation not only provides crucial resource allocation foundations and technological support for rural industrial integration but also drives modernization upgrades across industries through diversified development pathways of rationalization, sophistication, and advancement, enabling China’s economy to maintain competitiveness and stability in both domestic and international markets. Based on this analysis, the paper proposes the following hypothesis:

H2: Rural industrial integration can effectively enhance agricultural resilience through industrial structure transformation.

The rapid development of urbanization has profoundly transformed China’s economic structure and social configuration, serving as a critical engine driving economic growth, optimal resource allocation, and social structural transformation [54]. Traditional urbanization processes are characterized primarily by population migration and changes in land utilization patterns [55], fostering profound urban-rural interactions through multiple mechanisms including resource reallocation, market demand expansion, and technological diffusion [56,57]. This process has not only generated extensive economic growth and social transformation but has also influenced rural industrial integration to varying degrees. In the short term, urbanization facilitates rational allocation of human resources, market scale expansion, and gradual penetration of advanced technologies. However, in the long term, the massive influx of rural labor into urban areas results in human capital insufficiency and rising costs. Furthermore, during the urbanization process, supply pressure on land and natural resources intensifies progressively, constraining rural industrial integration from a resource foundation perspective. Airong [58] indicates that urbanization-induced urban population growth drives diversification and premium positioning of agricultural product demand, thereby generating significant changes in urban market demand structures. However, this transformation may expose agriculture to heightened market volatility risks, compelling the agricultural sector to struggle with maintaining stability and sustainable development while responding to urban market demands. Accordingly, Chavas [59] emphasizes that technological dissemination and application tend to prioritize high-output and capital-intensive industrial and service sectors, while the agricultural sector demonstrates certain disadvantages in technological adaptability and application capabilities. In summary, although urbanization generates high output value and substantial returns in the short term, it may exacerbate resource scarcity and structural imbalances in rural industries regarding rural development, thereby undermining agricultural resilience. Given these considerations, this study proposes the following hypothesis:

H3: Expansionary urbanization development will weaken the effect of rural industrial integration on enhancing agricultural resilience.

The inherent vulnerability and complexity of agriculture manifest as dynamic differences across regions with varying backgrounds in terms of resource endowment, economic development levels, market demand structures, socio-cultural and labor force structures, and ecological environments [45,60–63]. Simultaneously, constrained by the dynamic evolution of resource endowment, economic development levels, market environments, and policy orientations, rural industry integration demonstrates development patterns characterized by distinct regional features. These include market-oriented diversified integration in eastern regions, resource-oriented vertical integration in central regions, ecology-oriented specialized integration in western regions, and scaled agricultural modernization integration in northeastern regions [64–67]. Furthermore, the rural industry integration model in China’s major grain-producing regions primarily revolves around the grain industry chain, with grain production as the core, enhancing value chains through the development of grain processing and logistics industries. In contrast, non-major grain-producing regions focus on agro-cultural-tourism integration, branding and e-commercialization, and circular agriculture with ecological services [68]. Evidently, this complex and diverse interwoven pattern determines the behavioral diversity of agriculture when confronting external shocks. Based on this analysis, this study proposes the following hypothesis:

H4: Rural industry integration’s enhancement of agricultural resilience exhibits significant heterogeneity across economic zones and functional regions.

Rural industry integration involves interactive development among different regions, with “enclave economy” serving as a typical example [69]. Rural industry integration encompasses cross-sectoral integration of agriculture, processing industries, and service sectors, typically driving rural economic development through resource integration, industrial chain extension, and value addition enhancement. This process resembles “core-periphery” factor reorganization, where high-value-added industries (such as agricultural product processing, logistics, and e-commerce) form the “core,” driving the concentration and efficient utilization of agricultural resources. New economic geography demonstrates that regional economic development involves complex interactive mechanisms. When resource allocation and market demand in geographical space are interconnected, economic activities exhibit externalities, and inter-regional factor flows and market competition generate spillover effects [39], manifesting as the “Core-Periphery Model.” Generally, regions with relatively higher economic development levels tend to demonstrate greater vitality in industrial integration. Their advanced industrial models and technological expertise may spillover to neighboring regions through spatial economic proximity, generating “demonstration effects” or “radiation effects.” These effects manifest specifically through cross-regional resource allocation optimization, cross-regional knowledge diffusion, cross-regional market demand expansion, and policy and economic environment coordination. For instance, China’s “Chengdu-Chongqing” economic circle, serving as a crucial regional development strategy to balance economic disparities between eastern and western regions, has promoted diversified rural economic development through the integration of primary, secondary, and tertiary industries [45]. Evidently, inter-regional socioeconomic activities typically exhibit significant spatial correlation. Based on this analysis, this study proposes the following hypothesis:

H5: Rural industry integration demonstrates significant spatial spillover effects in the process of enhancing agricultural resilience.

## 4. Methodology

### 4.1 Data sources

This study selects panel data from 31 provincial administrative regions in China (excluding Hong Kong, Macao, and Taiwan) spanning the period from 2012 to 2022 as observations. To address potential bias from missing data, this research employs linear interpolation methods to systematically complete missing values and applies 1% smoothing treatment to data with smaller numerical ranges in the panel, ensuring model stability and result rigor. The data are primarily sourced from the China Agricultural Yearbook, China Population and Employment Statistics Yearbook, China Rural Statistical Yearbook, China Rural Management Statistical Annual Report, EPS database, and local government documents..

### 4.2 Variables and data description

#### 4.2.1 Dependent variable.

Agricultural Resilience (AR). Based on resilience theory, this study follows Zampieri [25] in conceptualizing agricultural resilience as comprising resistance capacity, recovery capacity, and reconstruction capacity, adhering to the systematic cycle of disaster-recovery-reconstruction. Existing research on the resistance dimension focuses on the agricultural system’s initial capacity to withstand external shocks, encompassing production resilience, ecological resilience, and economic resilience.Production resilience indicators (total agricultural machinery power, original value of rural fixed assets, and irrigation rate) reflect hardware infrastructure support capacity; ecological resilience indicators (soil erosion control, pesticide usage, and disaster rate) demonstrate ecological environmental buffering capacity; while economic resilience indicators (per capita added value of agriculture, forestry, animal husbandry and fishery, per capita primary sector GDP, and rural living standards) quantify the system’s economic shock resistance capacity [26,45]. However, from the perspective of new institutional economics and rural development theory, this study identifies that the population base, knowledge reserves, and individual health of rural practitioners are closely interdependent with China’s rural social system. To comprehensively measure the overall level of agricultural resilience, this study incorporates “social resilience” following Qamar [70], specifically selecting “original value of productive fixed assets in rural household social services,” “number of medical facility beds per 10,000 rural population,” and “rural per capita education level” as proxy variables. The recovery capacity dimension scientifically evaluates the speed and ability of agricultural systems to restore their original functions and structures after experiencing shocks through recovery resilience indicators (rural employment structure, rural electricity efficiency, and per capita agricultural insurance premiums), reflecting the agricultural system’s self-repair potential and the sophistication of socioeconomic support networks [29]. The reconstruction capacity dimension comprehensively measures the agricultural system’s ability to achieve transformation and upgrading through technological innovation and knowledge accumulation after shocks via innovation resilience indicators (agricultural R&D investment intensity, number of agricultural technical personnel, and number of agricultural technology patents), reflecting the potential driving force for long-term adaptive development and structural optimization of agricultural systems, collectively constituting the dynamic development dimension of agricultural resilience evaluation [30] (see Table 1).

**Table 1:** Agricultural resilience indicator system.

Variable Name	Primary Indicator	Secondary Indicator	Indicator Explanation	Attribute	Weight
AR	Resistance	Production resilience	Agricultural machinery power (W)	+	0.301
			Original value of productive fixed assets in rural households (CNY)	+	0.349
			Efficiency of crop yield per unit area (%)	+	0.351
		Ecological Resilience	Soil erosion control area (M²)	–	0.265
			Pesticide usage (g)	+	0.339
			Agricultural disaster recovery rate (%):Ratio of actual disaster-affected agricultural area to total disaster-stricken agricultural area	–	0.395
		Economic Resilience	Per capita value-added of agriculture, forestry, animal husbandry, and fishery (CNY/person): Agricultural value-added/ Number of agricultural employees	+	0.309
			Per capita primary production GDP (CNY/m²): Total agricultural production/ Rural population	+	0.349
			Rural living standards (%): Rural residents’ per capita disposable income/ Per capita consumption expenditure	+	0.342
		Social Resilience	Original value of productive fixed assets in rural household social service industries (CNY/household)	+	0.356
			Number of hospital beds per 10,000 rural residents (units)	+	0.172
			Per capita rural education level (years)	+	0.471
	Recovery Capacity	Recovery Resilience	Rural employment structure (%): Number of employees in agriculture, forestry, animal husbandry, and fishery/ Total rural employment	+	0.336
			Rural electricity efficiency (W)	+	0.332
			Per capita agricultural insurance premium (CNY/person): Agricultural insurance income/ Number of employees in agriculture, forestry, animal husbandry, and fishery	+	0.332
	Regeneration	Innovation Resilience	Intensity of agricultural R&D funding (%)	+	0.375
			Number of agricultural technicians in collective enterprises and public institutions (persons)	+	0.355
			Number of agricultural technology patents (units)	+	0.269

#### 4.2.2 Explanatory variables.

Rural Industrial Integration (RII). Based on industrial integration theory and value chain integration perspectives, this study comprehensively evaluates agricultural industrial diversification development across five dimensions: agricultural industrial chain extension (eaic), agricultural multifunctional expansion (mea), integration of agriculture and service industry (iasi), cultivation of new agricultural business forms (cna), and improvement of interest linkage mechanisms (iblm) (Table 2). Among these dimensions, agricultural industrial chain extension measures the depth of agricultural extension into downstream industries through three aspects: processing industry level, manufacturing industry level, and sales capacity [71] Agricultural multifunctional expansion assesses the diversification degree of agricultural functions from the perspectives of ecological efficiency, environmental improvement, and rural tourism [72]. The cultivation of new agricultural business forms measures the development level of modern agricultural formats through facility agriculture level, large-scale new business form support, and water-saving agriculture depth [73]. The integrated development of agriculture and service industry reflects the degree of integration between productive service industries and agriculture across three dimensions: climate services, mechanical services, and logistics systems [74].

**Table 2:** Rural industrial integration.

Variable Name	Primary Indicator	Secondary Indicator	Indicator Explanation	Attribute	Weight
Rural industrial integration	Agricultural Industrial Chain Extension	Level of Agricultural Processing Industry	Number of Newly Established Agricultural Processing Enterprises (units)	+	0.317
		Level of Agricultural Manufacturing Industry	Original Value of Manufacturing Fixed Productive Assets Owned by Rural Households (CNY/household)	+	0.342
		Capacity for Agricultural Product Sales	Original Value of Wholesale and Sales Fixed Productive Assets Owned by Rural Households (CNY/household)	+	0.340
	Agricultural Multifunctional Expansion	Agricultural Ecological Efficiency	Composting and Harmless Disposal Capacity of Household Waste (kg/day)	+	0.413
		Rural Ecological Improvement Status	Carbon Emissions from Tillage (10,000 tons)	+	0.328
		Level of Rural Tourism Expansion	Demonstration Sites for Recreational Agriculture and Rural Tourism (units)	+	0.259
	Cultivation of New Agricultural Business Models	Level of Facility Agriculture	Facility Agriculture Total Area/ Total Cultivated Land Area (%)	+	0.389
		Support for Large-Scale New Business Models	Organic Food Certifications (units)	+	0.334
		Level of Water-Saving Agriculture	Agricultural Plastic Film Usage (kg)	+	0.277
	Integration of Agricultural Services Development	Level of Climate Services	Agricultural Meteorological Observation Service Stations (units)	+	0.331
		Agricultural Machinery Services	Number of Agricultural Machinery Service Organizations (units)	+	0.358
		Rural Logistics System	Rural Delivery Routes (km)	+	0.311
	Improvement of Benefit-Linkage Mechanisms	Level of Organizational Cooperation	Number of Farmer Cooperatives (units)	+	0.305
		Agricultural Risk Sharing	Agricultural Insurance Density (CNY/person)	+	0.367
		Agricultural Industrial Business Entities	Number of Leading Agricultural Enterprises (units)	+	0.327

#### 4.2.3 Control variables.

Following Liang [75], this study selects digitalization level (Digital), infrastructure level (transportation network density IL), and per capita GDP (GDP) as control variables to effectively control for the potential impacts of inter-regional differences in digital capabilities, infrastructure, and economic development levels on agricultural resilience, thereby ensuring the robustness of empirical results and the scientific validity of theoretical interpretations.

#### 4.2.4 Mediating variables.

Structural Change Index (SCI).Structural change refers to the process of resource allocation optimization and functional upgrading among primary, secondary, and tertiary industries within an economic system, manifesting as the dynamic evolution of industrial structure rationalization (improved resource allocation efficiency), sophistication (deepened division of labor), and advancement (industrial modernization and high-end development). Therefore, drawing on Moore’s [76] classical industrial structure theory and Rostow’s [77] stages of economic development theory, this study constructs a multidimensional measurement system for structural change, aiming to provide a new analytical perspective for understanding the internal mechanisms of agricultural resilience enhancement (Table 3).

**Table 3:** Mediating variable.

Variable Name	Primary Indicator	Secondary Indicator	Indicator Explanation	Attribute	Weight
Industrial Structure Change	Industrial Structure Rationalization	Industrial Synergy Development	Theil Index (Gu, 2022)	–	0.155
	Industrial Structure depth	Industrial Share	Primary Industry/GDP × 1 + Secondary Industry/GDP × 2 + Tertiary Industry/GDP × 3 (Fu, 2024)	+	2.375
	Industrial Structure advanced	Industrial Technological Innovation	Secondary Industry Output/ Tertiary Industry Output (Cheng, 2018)	+	1.231

#### 4.2.5 Moderating variables.

Urbanization Level (UL).Urbanization level refers to the degree of population and economic activity concentration toward urban areas within a country or region, typically measured by the proportion of urban population to total population or other indicators such as urban construction land area and urban functional completeness [78]. However, traditional indicator measurements are often constrained by limitations of the household registration system, making it difficult to comprehensively reflect the actual situation of population mobility. Following Anderson [79], this study adopts “(resident population - registered population)/resident population” as the measurement indicator for urbanization level, which not only dynamically captures the migration trends of agricultural transfer population but also reveals the gap between “population urbanization” and “hukou urbanization.” Compared to traditional methods, the urbanization level captures the differential impacts of rural industrial integration on agricultural resilience under varying urbanization contexts, revealing the critical role of urbanization in promoting optimal allocation of urban-rural resources, market linkage, and labor force transfer.

### 4.3 Model design

To avoid the randomness of subjective weighting and ensure the credibility and scientific validity of indicator weights, this study follows Chen [80] and employs the entropy weighting method using Python 3.11.5 to determine indicator weights. The specific steps are as follows: Select m provinces and n indicators, where $x_{ij}$ represents the value of the j-th indicator for the i-th province (i=1,2,3…,m;j=1,2,3….,n).

First, data standardization is performed to eliminate the impact of dimensionality and positive/negative orientations on evaluation results. The formula for processing positive indicators is:


$$y_{ij}=\frac{x_{ij}-min(x_{1j},x_{2j},\ldots ,x_{mj})}{\max\left(x_{1j,x_{2j},\ldots ,x_{mj}}\right)-min(x_{1j},x_{2j},\ldots ,x_{mj})}$$ (1)

The formula for processing negative indicators is:


$$y_{ij}=\frac{max\left(x_{1j},x_{2j},\ldots ,x_{mj}\right)-x_{ij}}{\max\left(x_{1j,x_{2j},\ldots ,x_{mj}}\right)-min(x_{1j},x_{2j},\ldots ,x_{mj})}$$ (2)

Second, calculate the proportion of the j-th indicator for the i-th province:


$$x_{ij}=\frac{y_{ij}}{\sum_{i=1}^{m}y_{ij}}$$ (3)

Third, calculate the information entropy of the j-th indicator:


$$\begin{array}{c} \;z_{j}=-\frac{1}{lnm}\sum {\mathrm{\backslash nolimits}}_{i=1}^{m}x_{ij}\times \ln x_{ij},0\leq z_{j}\leq 1 \end{array}$$ (4)

Fourth, calculate the information redundancy of the j-th indicator:


$$e_{j}=1-z_{j}$$ (5)

Fifth, calculate the indicator weights:


$$w_{j}=\frac{e_{j}}{\sum_{j=1}^{n}e_{j}}$$ (6)

Sixth, calculate the comprehensive score:


$$\begin{array}{c} s_{j}=\sum {\mathrm{\backslash nolimits}}_{j=1}^{n}w_{j}\times y_{ij} \end{array}$$ (7)

(1) Kernel Density Distribution. Considering that both rural industrial integration and agricultural resilience exhibit characteristics of wide distribution, long cycles, and high volatility, scholars cannot identify potential patterns or trends through smooth probability distributions to reveal the concentration or dispersion of certain phenomena in geographical or social structures. Therefore, based on the constructed indicator system, this study follows Parzen’s [81] research paradigm and employs the Gaussian Function to obtain the kernel density distributions of both variables. This approach facilitates comprehensive and multi-perspective demonstration of the dynamic evolutionary distribution, underlying patterns, and identification of “hotspots” (high-value clustering areas) and “cold spots” (low-value clustering areas) across 31 provincial administrative regions from 2012 to 2022, with calculation formulas shown in equations (8) and (9):


$$\begin{array}{c} f(x)=\frac{1}{Nh}\sum {\mathrm{\backslash nolimits}}_{i=1}^{N}K\left(\frac{X_{i}-\bar{x}}{h}\right) \end{array}$$ (8)

$$\begin{array}{c} K(x)=\frac{1}{\sqrt{2\pi }}exp\left(-\frac{x^{2}}{2}\right) \end{array}$$ (9)

Here, f(·) represents the density function, K(·) is the kernel function, N is the number of observations, $x_{i}$ denotes the observed values, $\frac{-}{x}$ is the mean, and h is the bandwidth.

(2) Basic Regression Model. Following Tian’s [82] research design, based on direct effects analysis, rural industrial integration is divided into agricultural industrial chain extension (eaic), agricultural multifunctional expansion (mea), integration of agriculture and service industry (iasi), cultivation of new agricultural business forms (cna), and improvement of interest linkage mechanisms (iblm):


$${AR}_{it}=\beta_{0}+\beta_{1}{RII}_{it}+\beta_{1}{DIGITAL}_{it}+\beta_{2}{IL}_{it}+\beta_{3}{GDP}_{it}+\varepsilon_{it}$$ (10)

$$\begin{array}{c} {AR}_{it}=\beta_{0}+\beta_{1}{eaic}_{it}+\beta_{2}{mea}_{it}+\beta_{3}{iaisi}_{it}+\beta_{4}{cna}_{it}+\beta_{5}{iblm}_{it}+\beta_{6}{DIGITAL}_{it}+\beta_{7}{IL}_{it}+\beta_{8}{GDP}_{it}+\varepsilon_{it} \end{array}$$ (11)

(3) Mediation Effects. Following Dowd [83], this study selects structural change (industrial rationalization, sophistication, and advancement) to further reveal the transmission mechanism through which rural industrial integration (RII) enhances agricultural resilience (AR) via structural change (SCI):


$${AR}_{it}=\beta_{0}+\beta_{1}{RII}_{it}+\varepsilon_{it}$$ (12)

$${RII}_{it}=\beta_{0}+\beta_{1}{SCI}_{it}+\varepsilon_{it}$$ (13)

$${AR}_{it}=\gamma_{0}+\gamma_{1}{SCI}_{it}+\gamma_{2}{RII}_{it}+\varepsilon_{it}$$ (14)

(4) Moderation Effects Model. Following Guan [54], this study verifies the dynamic moderating effects of urbanization level (UL) on the relationship between agricultural industrial integration (AII) and agricultural resilience (AR), establishing the following econometric model:


$${AR}_{it}=\beta_{0}+\beta_{1}{AII}_{it}+\varepsilon_{it}$$ (15)

$${AR}_{it}=\beta_{0}+\beta_{1}{AII}_{it}+\beta_{2}{UL}_{it}+\varepsilon_{it}$$ (16)

$${AR}_{it}=\beta_{0}+\beta_{1}{AII}_{it}+\beta_{2}{UL}_{it}+\beta_{3}({AII}_{it}\times {UL}_{it})+\varepsilon_{it}$$ (17)

(5) Spatial Model Construction:。Traditional economist Adam Smith emphasized the importance of division of labor and productivity in enhancing national wealth levels in his 1776 work “An Inquiry into the Nature and Causes of the Wealth of Nations,” while per capita GDP, as an indicator of per capita output value, precisely reflects the productivity level of a country or region [84].Research indicates that when the per capita GDP difference between two regions is substantial, it signifies a significant gap in their economic development levels. Based on this research characteristic, differences in economic levels significantly influence the outcomes of rural industrial integration and agricultural resilience. Therefore, this study selects “per capita GDP difference” as matrix W to construct a spatial economic matrix model.

Exploring whether significant spatial interaction relationships exist between rural industrial integration and agricultural resilience constitutes a crucial component in revealing regional development patterns and formulating targeted policies. In this process, testing spatial correlation represents an indispensable key step. In spatial correlation analysis, the Global Moran’s I index, as the most classical and commonly used method, can quantify the spatial clustering characteristics of inter-regional variable distributions, providing an intuitive global perspective for research. The model is shown as follows:


$$\;I=\frac{\sum_{i=1}^{n}\sum_{j=1}^{n}W_{it}\left(X_{i}-\bar{X}\right)\left(X_{j}-\bar{X}\right)}{S^{2}\cdot \sum_{i=1}^{n}\sum_{j=1}^{n}W_{ij}}$$ (18)

In the formula, n represents the total number of spatial units, $X_{i}$ and $X_{j}$ denote the attribute values of the random variable X in geographic units i and j, respectively, and $\bar{X}$ is the mean of the attribute values across n spatial units. $S^{2}=\frac{\sum_{i=1}^{n}{\left(X-\bar{X}\right)}^{2}}{n}$ represents the sample variance, $W_{ij}$ is the (i,j)-th element of the spatial weight matrix (used to measure the distance between region i and region j), and $\sum_{i=1}^{n}\sum_{j=1}^{n}W_{it}$ is the sum of spatial weights. The global Moran’s I takes values between 0 and 1 for positive spatial correlation, indicating that similar attributes cluster together; values between −1 and 0 indicate negative spatial correlation, where dissimilar attributes cluster together; values close to 0 suggest random distribution or the absence of spatial autocorrelation.

Spatial Weight Matrix. This study considers that the spillover effects of rural industrial integration on agricultural resilience may vary under different spatial factors, and the weight matrix is selected as follows:


$$W_{ij}=\{\begin{array}{c} \frac{1}{|{\overset{―}{y}}_{i}-{\overset{―}{y}}_{j}|,i\neq j}\\ 0,i=j \end{array}$$ (19)

In the formula, ${\overset{―}{y}}_{i}$ represents the per capita GDP of region i, and ${\overset{―}{y}}_{j}$ represents the per capita GDP of region j. The economic distance matrix in this study is based on the reciprocal of the difference in the average per capita GDP between two regions from 2012 to 2022, used as an indicator to measure economic distance.

Spatial Panel Models. When analyzing the spatial correlations of regional economic development or policy effects, spatial panel models serve as essential analytical tools, with their core strength lying in characterizing the complex spatial interaction mechanisms among variables. The primary forms of these models include the Spatial Error Model (SEM), Spatial Autoregressive Model (SAR), and Spatial Durbin Model (SDM). Among these, the Spatial Durbin Model demonstrates significant advantages by simultaneously incorporating both endogenous and exogenous spatial interaction effects, whereas the Spatial Autoregressive Model focuses exclusively on endogenous spatial interactions, and the Spatial Error Model emphasizes the spatial correlation characteristics of error terms.Under specific conditions, the Spatial Durbin Model can be simplified to a Spatial Error Model, and this flexibility further demonstrates its broad applicability.Based on the characteristics of this research, this study ultimately selects the Spatial Durbin Model (SDM) to conduct empirical analysis, with the specific model specification presented as follows:

Spatial Panel Model. In analyzing the spatial correlation of regional economic development or policy effects, the spatial panel model has become an important tool, with its core focus on depicting the complex spatial interaction mechanisms among variables. The main forms of this model include the Spatial Error Model (SEM), the Spatial Autoregressive Model (SAR), and the Spatial Durbin Model (SDM). Among them, the Spatial Durbin Model (SDM) has significant advantages as it incorporates both endogenous and exogenous spatial interaction effects, whereas the Spatial Autoregressive Model (SAR) focuses only on endogenous spatial interactions, and the Spatial Error Model (SEM) emphasizes the spatial correlation of error terms. Under certain conditions, the Spatial Durbin Model can be simplified into the Spatial Error Model, further highlighting its versatility and broad applicability. Based on the research characteristics, this paper ultimately adopts the Spatial Durbin Model (SDM) for empirical analysis. The specific form of the model is shown as follows:


$$\;Y_{it}=\rho W_{j}Y_{it}+\beta_{1}X_{it}+\theta_{1}W_{j}X_{it}+\mu_{t}+\varepsilon_{it}$$ (20)

In the formula, i and t represent provinces and time, respectively; other variable definitions are consistent with Equations (18)-(20); $W_{j}$ represents the spatial weight matrix, where j=1denotes the economic weight matrix. ρ is the spatial autoregressive coefficient, which primarily reflects the spatial spillover effect. $\beta_{1}$ represents the effect of the explanatory variable in the local region on the dependent variable in the same region. $\theta_{1}$ is the coefficient of the spatial lagged variable. When $\theta_{1}$>0, it indicates a positive spillover effect of the explanatory variable in the local region on the dependent variable in neighboring regions’ economic development. Conversely, when $\theta_{1}$<0, it indicates a negative spillover effect.

## 5. Results and discussion

This study systematically analyzed the internal mechanisms between rural industrial integration and agricultural resilience using Eviews13, STATA, and MATLAB software. First, correlation analysis reveals that the correlations between variables generally range from 0.3 to 0.5 (< 0.7), indicating moderate linear correlations among the data. Second, the Mean VIF value of 4.97 (< 10) suggests that no severe multicollinearity issues exist among the variables in the baseline regression model, thereby demonstrating variable independence and model validity from a statistical perspective.

### 5.1 Kernel density trend analysis

**Fig 3:**
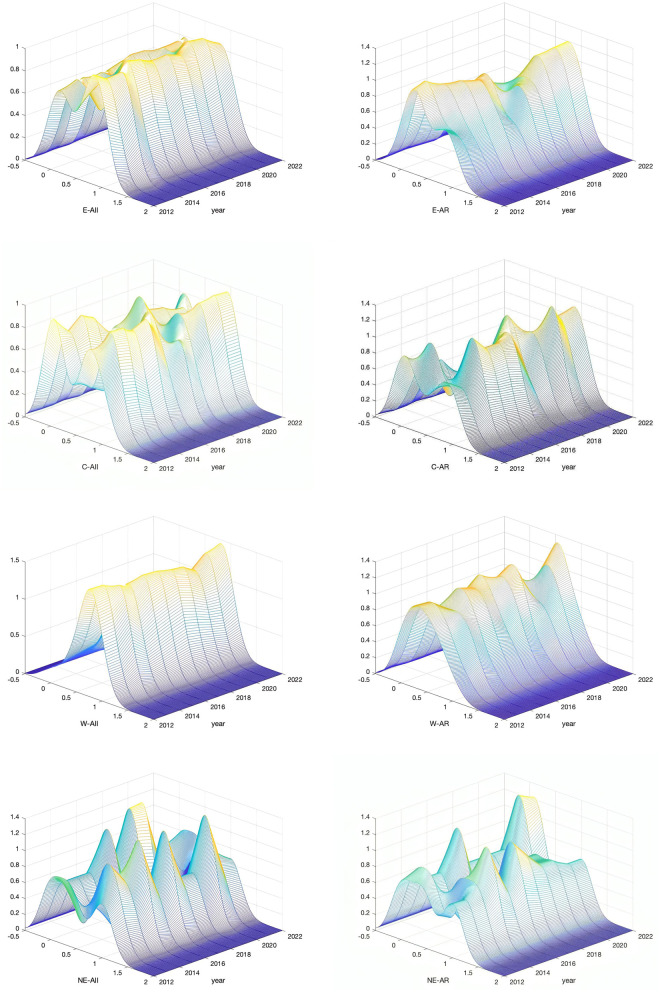
Regional heterogeneity of the kernel index.

This study employs kernel density estimation methods to generate visualization curves, demonstrating the evolutionary trajectories of rural industrial integration and agricultural resilience from perspectives of distributional patterns, distributional characteristics, distributional extensibility, and polarization features (see Fig 3). The findings reveal that rural industrial integration in the eastern region increased from 0.380 in 2012 to 0.760 in 2022, achieving a remarkable 100% growth over the decade with an average annual growth rate of approximately 3.9%. During 2012–2016, index growth remained relatively moderate with an average annual growth rate of approximately 3.2%, indicating that policy effects were beginning to emerge during this period, though rural industrial chains remained predominantly focused on traditional cultivation and primary processing. From 2016–2022, benefiting from the rapid emergence of new business formats such as rural e-commerce, agricultural processing, and leisure agriculture in the eastern region, the average annual growth rate accelerated to 4.4%. From 2012 to 2022, rural industrial integration in the central region increased from 0.538 to 0.707, representing an overall growth of 31.4% with an average annual growth rate of approximately 2.6%. Data analysis indicates that 2016 served as a turning point, coinciding with the implementation of national policies such as “supply-side structural reform” and “rural revitalization strategy,” demonstrating that policy orientation played a crucial role in regional transformation. From 2012 to 2022, the rural industrial integration index in the western region increased from a minimum of 0.248 to a maximum of 0.662, achieving an overall growth of 166.5% with an average annual growth rate of approximately 3.0%. The most significant growth occurred in 2015, with the most substantial index increase of 0.045 and a growth rate of 9.03%, far exceeding the growth rates of other years. This growth was primarily attributed to the vigorous promotion of farmer production cooperatives and significant progress in the integrated development of agriculture with eco-tourism and e-commerce platforms. However, from 2016 to 2018, growth rates significantly decelerated, with average annual increases below 0.5%, indicating that certain regions entered phases of resource integration and model optimization following their development stages. Given that the northeastern region has historically focused on traditional agriculture, with rural industrial integration primarily concentrated in sectors such as cultivation, primary processing, and manufacturing with relatively low added value, the average annual growth rate of the rural industrial integration index in the northeastern region reached only 2.3%. Even during the period of rapid national economic growth from 2016–2022, the industrial integration rate in the northeastern region reached only 2.7%, demonstrating insufficient development momentum. The principal factors contributing to low industrial integration in the northeastern region include limited industrial diversification, insufficient regional coordination, inadequate investment in technology and capital, and high external dependency.

Over the past decade, the agricultural resilience index in the eastern region has demonstrated a sustained upward trend, achieving an overall growth of 86.2% with an average annual growth rate of approximately 3.5%. During 2012–2016, the index growth remained relatively moderate with an average annual growth rate of 3.2%. However, from 2016–2022, the growth rate accelerated significantly, reaching an average annual growth rate of 4.0%. Notably, after 2016, the kernel density curve shifted rightward with a more compact distribution, indicating that regional disparities in agricultural resilience gradually diminished and regional development became more balanced. The eastern region’s advanced logistics systems, markets, and superior resource endowments have established a solid foundation for comprehensive agricultural resilience improvement. Particularly, the construction of national “smart agriculture demonstration parks” and “high-standard farmland construction projects” has facilitated the rapid development of new models such as rural e-commerce and cold-chain logistics, thereby enhancing the risk resistance capacity of agricultural industrial chains in the eastern region. The agricultural resilience level in the central region has steadily increased with a growth rate of approximately 42.8% and an average annual growth rate of 3.3%. Specifically, during 2012–2016, the index showed modest growth with an average annual growth rate of 2.8%, while from 2016–2022, growth accelerated with the average annual growth rate increasing to 3.8%. Taking Henan Province, known as the “Central Plains Granary,” as an example, recent years have witnessed comprehensive industrial chain extension through industrial structure optimization, including agricultural product processing, e-commerce, leisure agriculture, and ecological agriculture. The western region’s agricultural resilience has demonstrated exceptional performance, with an overall growth rate of approximately 124.5% and an average annual growth rate of 4.1%. During this period, the agricultural resilience index showed modest growth from 2012–2016 with an average annual growth rate of 3.2%, which increased to 4.6% from 2016–2022. Research findings indicate that after 2016, western agricultural resilience concentrated in high-value areas, though these regions remained geographically dispersed, demonstrating significant unbalanced growth patterns. This phenomenon primarily stems from the western region’s abundant land resources and ecological diversity, which provide inherent advantages for developing specialized agriculture such as traditional Chinese medicinal herb cultivation, grassland animal husbandry, and specialty fruit production. It should be noted that due to complex natural conditions and weak infrastructure, improving agricultural resilience requires sustained long-term investment. Agricultural resilience in the northeastern region improved by approximately 33.7% during 2012–2022. During 2012–2016, agricultural resilience growth remained slow with an average annual growth rate of only 1.5%. With policy optimization and gradual promotion of modern agricultural technologies, the average annual growth rate has increased to 2.8%. As a major grain-producing region, the northeast possesses high-quality “black soil” resources. However, this advantage continues to face significant challenges from arable land degradation and climate change. Consequently, the government has implemented policies of “storing grain in land and technology” and the “food security strategy,” providing solid guarantees for improving agricultural resilience in the northeastern region. Nevertheless, compared to the complex risks faced and development requirements, the overall improvement of agricultural resilience in the northeastern region remains insufficient.

### 5.2 Baseline regression model

Table 4 presents the sensitivity analysis of rural industrial integration and its multidimensional heterogeneity on agricultural resilience enhancement. The results indicate that the estimated coefficient of rural industrial integration (RII) is 0.491 and statistically significant at the 1% level, providing preliminary evidence that rural industrial integration has a positive impact on agricultural resilience (Model 1). Considering the diversified nature of rural industrial integration, we further categorized it into agricultural industrial chain extension (eaic), agricultural multifunctional expansion (mea), agriculture-service industry integration (iasi), cultivation of new agricultural business models (cna), and improvement of benefit linkage mechanisms (iblm), with estimated coefficients of 0.175, 0.351, 0.075, 0.109, and 0.324, respectively. All dimensions are statistically significant at the 1% level except for the cultivation of new agricultural business models (cna) (Models 2–6). Within the unique context of China’s rural development, agricultural multifunctional expansion and the improvement of benefit linkage mechanisms are recognized as critical pathways for enhancing agricultural resilience, serving as key elements for income stability, risk diversification capacity, and social capital accumulation. The primary reason lies in their unique contributions to agricultural economic stability and sustainability. Agricultural multifunctional expansion, through models such as agritourism, ecological agriculture, and circular agriculture, provides farmers with diversified income sources. This approach not only effectively disperses natural and market risks but also generates compounded ecological and social benefits, significantly enhancing the environmental adaptability of agricultural systems. Benefit linkage mechanisms, by optimizing the distribution relationships between farmers and enterprises or cooperatives, significantly enhance the stability of farmers’ income and the organizational level of the agricultural economy. Mechanisms such as contract farming, cooperative models, and profit-sharing systems provide farmers with effective income security, reducing the impact of market volatility on farmers’ earnings. Consistent with these findings, Darnhofer [85], using Austria as a case study, confirmed the continued applicability of multifunctional agricultural management in developed countries. However, Western contract farming focuses primarily on market-oriented contractual relationships, emphasizing legal constraints and individual risk allocation, whereas China’s benefit linkage mechanisms place greater emphasis on “community” building, incorporating innovative models such as guaranteed procurement, risk sharing, and profit distribution [86,87].

**Table 4:** Basic regression model variable names.

Variable Name	Model 1	Model 2	Model 3	Model 4	Model 5	Model 6
*Rural Industrial Integration*	0.491*** (0.06)	0.175*** (0.04)	0.351*** (0.08)	0.075*** (0.02)	0.109* (0.06)	0.324*** (0.05)
*Digitalization Level*	0.028* (0.01)	0.023 (0.01)	0.033** (0.02)	0.028* (0.02)	0.031* (0.01)	0.017 (0.01)
*Transportation Network Density*	0.013*** (0.01)	0.009** (0.01)	0.016*** (0.01)	0.007 (0.01)	0.011* (0.01)	0.013*** (0.01)
*Per Capita GDP*	0.217*** (0.05)	0.251*** (0.05)	0.0198*** (0.05)	0.222*** (0.05)	0.202*** (0.05)	0.132*** (0.04)
*Robustness Test*	0.495*** (0.06)	0.167*** (0.05)	0.258*** (0.03)	0.592*** (0.28)	0.108* (0.06)	0.299*** (0.05)
*Individual Fixed Effects*	Yes	Yes	Yes	Yes	Yes	Yes
*Time Fixed Effects*	Yes	Yes	Yes	Yes	Yes	Yes
*Constant*	−0.917*** (0.23)	−0.831*** (0.24)	−0.812*** (0.25)	−0.661*** (0.24)	−0.615*** (0.24)	−0.415* (0.23)
*R* ^*2*^	0.943	0.938	0.939	0.937	0.937	0.944
*DWS*	1.618	1.523	1.602	1.891	1.241	1.412

The coefficients for agricultural industry chain extension, new business format cultivation, and agricultural service industry integration are relatively small, with new business format cultivation failing to reach even the 1% significance level. This stems from structural constraints in China’s industrial integration. Specifically, Wang [88] revealed that China’s agricultural product processing rate is approximately 67.5%, which is lower than the 85% level observed in developed countries. Additionally, the presence of “small, scattered, and weak” enterprises and poor industrial chain connectivity results in unstable contractual relationships between processing enterprises and farmers, thereby limiting the contribution of industry chain extension to agricultural stability. Furthermore, the inherent vulnerability of agriculture has led to a dual structure in China’s agricultural services characterized by “public service dominance and insufficient market services” [89]. While public services have broad coverage, they lack precision, and market-oriented services remain underdeveloped. This creates a mismatch between service supply and farmer demand, thereby weakening the enhancing effect of service integration on resilience. Moreover, Wu [90] empirically found that farmer participation in new business formats such as digital agriculture is only 18.6% in China, indicating a significant “digital divide.” These findings challenge Martin’s [91] emphasis in resilience evolution theory that industry chain integration serves as the core driving factor of agricultural resilience. However, the impact of organizational innovation on agricultural resilience often manifests before that of technological innovation. This development pattern of “organizational leadership followed by technological advancement” also applies to other developing countries [92]. In view of this, Hypothesis H1 is established.

### 5.3 Mediation effects

Table 5 demonstrates the mechanism and effects of the mediating variable (industrial structure transformation). Specifically, models 1–3 verified that rural industrial integration further enhances agricultural resilience through industrial structure rationalization, with significance at the 1% statistical level. Industrial structure rationalization not only improves the linkage between agriculture and other industries and enhances resource allocation efficiency, but also injects greater innovation vitality into agriculture, enabling it to demonstrate stronger adaptability and risk resistance when facing external uncertainties. In contrast, models 4–6 and models 7–9 found that rural industrial integration fails to effectively enhance agricultural resilience through industrial structure sophistication and advancement, and even demonstrates attenuation at the 1% statistical level.

**Table 5:** Mediating effect model.

Variable Name	Model 1	Model 2	Model 3	Model 4	Model 5	Model 6	Model 7	Model 8	Model 9
	Rationalization of Industrial Structure			industrial structure depth			advanced of industry structure		
*Rural Industrial Integration*	0.491*** (0.06)	0.138** (0.09)	0.495*** (0.06)	0.491*** (0.06)	−0.181*** (0.06)	0.465*** (0.06)	0.491*** (0.06)	−3.288*** (0.43)	0.379*** (0.06)
*Mediating Effect*			0.174*** (0.04)			−0.141*** (0.05)			−0.034*** (0.01)
*Digitalization Level*	0.028* (0.01)	0.013 (0.03)	0.027* (0.01)	0.028* (0.01)	0.104*** (0.02)	0.043*** (0.02)	0.028* (0.01)	0.358*** (0.11)	0.041*** (0.01)
*Transportation Network Density*	0.013*** (0.01)	−0.011** (0.01)	0.012*** (0.05)	0.013*** (0.01)	0.006 (0.01)	0.014*** (0.01)	0.013*** (0.01)	0.083*** (0.03)	0.016*** (0.01)
*Per Capita GDP*	0.217*** (0.05)	−0.350** (0.11)	0.194*** (0.05)	0.217*** (0.05)	−0.437*** (0.05)	0.156*** (0.05)	0.217*** (0.05)	−2.914*** (0.32)	0.119** (0.05)
*Robustness Test*	0.495*** (0.06)	−1.139*** (0.12)	0.679*** (0.21)	0.495*** (0.06)	−0.385*** (0.55)	0.407*** (0.06)	0.495*** (0.06)	−4.422*** (0.65)	0.609*** (0.04)
*Individual Fixed Effects*	Yes	Yes	Yes	Yes	Yes	Yes	Yes	Yes	Yes
*Time Fixed Effects*	Yes	Yes	Yes	Yes	Yes	Yes	Yes	Yes	Yes
*Constant*	−0.917*** (0.23)	0.942* (0.55)	−0.753** (0.22)	−0.917*** (0.23)	4.441*** (0.24)	−0.291 (0.32)	−0.917*** (0.23)	1.642*** (0.41)	−0.368 (0.26)
*R2*	0.943	0.932	0.949	0.943	0.978	0.947	0.943	0.976	0.948
*DWS*	1.518	1.587	1.612	1.818	1.641	1.644	1.518	1.882	1.693
*Sobel Test*	1.987***			2.134***			2.256***		
*Indirect Effect*	0.0253								
*Proportion of Indirect Effect*	7.25								

The possible reason is that China’s rural industrial structure is singular, with excessive capital concentration in high-return sectors such as non-grain crop cultivation [93]. This highly concentrated resource allocation within a singular structure tends to weaken agriculture’s responsive capacity when confronting market fluctuations, policy adjustments, or sudden disasters. Additionally, Altieri [94] found that rural industrial structure sophistication is often accompanied by industrial disorder. Governments blindly pursue performance evaluations without distinction, unilaterally seeking short-term fiscal revenue while over-relying on investment attraction and rapid implementation of industrial projects (such as high-end agricultural product processing), neglecting subsequent maintenance costs, ongoing expenses, and inter-departmental coordinated development. Such behavior not only leads to unreasonable resource utilization but also creates imbalances in the relationship between agriculture and other industries, further amplifying the vulnerability of rural economic structures. Consistent with this, there is a pronounced mismatch between agricultural modernization technology promotion and farmers’ skill levels, with numerous farmers unable to integrate into modern agricultural industry chains due to lack of professional skills training, resulting in human capital constraints becoming a bottleneck for industrial upgrading. Furthermore, excessive emphasis on high-tech industrial park construction tends to neglect traditional aspects of rural agriculture (such as land protection, infrastructure improvement, and low-technology agricultural development). This development model of “emphasizing high-end while neglecting fundamentals” exacerbates the dual differentiation of rural industrial structures, constraining the quality and sustainability of industrial structure advancement. In contrast, Swinnen [95] found in European and American agriculture that industrial structure advancement typically correlates positively with agricultural resilience, reflecting structural differences between China’s smallholder economy and the large-scale agriculture of developed countries. However, in African countries, excessive industrial concentration may lead to the marginalization of small-scale producers and increase systemic vulnerability, reinforcing the common characteristics of agricultural resilience construction in developing countries [96].

To verify the reliability of the statistical results, this study employs the Sobel test to examine the mediating effects of industrial structure transformation. The Sobel test evaluates the statistical significance of mediation pathways by calculating the standard errors of indirect effects, with the test statistic following a standard normal distribution. First, industrial structure rationalization. The path coefficient from rural industrial integration to industrial structure rationalization is 0.138 (p < 0.05), while the path coefficient from industrial structure rationalization to agricultural resilience is 0.174 (p < 0.01), yielding a Sobel statistic of 1.987 (p < 0.01). Second, industrial structure intensification. The corresponding path coefficients are −0.181 and −0.141, respectively, with a Sobel statistic of 2.134 (p < 0.01). Third, industrial structure upgrading, with corresponding path coefficients of −3.288 and −0.034, and a Sobel statistic of 2.256 (p < 0.01). All Sobel test values are significant at the 1% level, indicating that rural industrial integration exerts robust indirect effects on industrial structure transformation through mediating variables. These findings suggest that during rural industrial integration, particular attention should be paid to the rational configuration of industrial structure, while carefully advancing the processes of industrial intensification and upgrading to ensure the sustained enhancement of agricultural resilience. Therefore, hypothesis H2 is partially supported.

### 5.4 Moderation effects

Table 6 presents the moderation effects results for urbanization level, measured as “(permanent residents - registered population)/permanent residents.” Model 1 shows that the baseline model coefficient is 0.491. After incorporating urbanization level in Model 2, the positive effect of rural industrial integration remains significant at 0.494, while urbanization level demonstrates a potentially inhibiting effect under specific conditions (−0.128). The interaction term in Model 3 reveals an estimated coefficient of −0.877, significant at the 1% level, indicating that urbanization level attenuates the positive effect of rural industrial integration on agricultural resilience enhancement.

**Table 6:** Moderating effect model.

Variable Name	Model 1	Model 2	Model 3
*Rural Industrial Integration*	0.491*** (0.06)	0.494*** (0.06)	0.481*** (0.06)
*Urbanization Level*		−0.128** (0.06)	0.367** (0.16)
*Interaction Term*			−0.877*** (0.28)
*Digitalization Level*	0.028* (0.01)	0.037** (0.02)	0.027* (0.01)
*Transportation Network Density*	0.013*** (0.01)	0.011** (0.01)	0.011** (0.01)
*Per Capita GDP*	0.217*** (0.05)	0.236*** (0.05)	0.210*** (0.05)
*Robustness Test*	0.495*** (0.06)	−0.143** (0.08)	−1.279*** (0.63)
*Individual Fixed Effects*	Yes	Yes	Yes
*Time Fixed Effects*	Yes	Yes	Yes
*Constant*	−0.917*** (0.23)	−0.994*** (0.23)	−0.811*** (0.23)
*R* ^*2*^	0.943	0.934	0.947
*DWS*	1.518	1.576	1.489

The potential reasons primarily stem from the paradox inherent in the urbanization process. The surge in urbanization levels and population mobility ratios highlights the attractiveness, economic vitality, and resource allocation efficiency of modern cities, but simultaneously signifies the departure of substantial numbers of young and middle-aged workers from agricultural production [97], resulting in rural labor imbalances. However, Promkhambut [98] observed in Thailand that rural labor migration did not lead to large-scale smallholder exit, with overall stable development maintained. Earlier research by García-Barrio [99] argued that agricultural production has long depended on collective forms such as cooperatives and household responsibility systems to achieve resource sharing and risk pooling. With substantial labor migration, traditional rural cooperative mechanisms have gradually disintegrated, making it difficult to sustain collective agricultural production methods and reducing resource-sharing efficiency [100]. Furthermore, urbanization has caused the gradual loosening of traditional community support networks based on geographical and kinship ties, with rural solidarity among farmers progressively weakening [101]. This finding resonates with Adger’s [102] conclusion from multiple global regions that community cohesion is positively correlated with disaster recovery capacity. Additionally, with the vigorous development of non-agricultural industries, particularly the rapid rise of real estate, service, and commercial sectors in urban peripheries, rural populations have been provided with more high-income employment opportunities, but this has simultaneously caused rural labor drain [103]. This phenomenon stands in stark contrast to the “return entrepreneurship” and “agricultural renaissance” trends observed by Dias [104] in developed countries, reflecting structural imbalances between China’s agricultural and urban industries in terms of attractiveness and resource allocation. Therefore, hypothesis H3 is supported.

### 5.5 Regional heterogeneity analysis

Table 7 reports that rural industrial integration exhibits regional heterogeneity in enhancing agricultural resilience. The eastern region demonstrates significant advantages in enhancing agricultural resilience due to superior policy frameworks, efficient integration models, and advanced technological conditions (Model 1). Similarly, the western region possesses excellent policy support and abundant resource advantages that unleash the potential for agricultural resilience enhancement (Model 3). Notably, rural industrial integration in the central and northeastern regions fails to effectively enhance agricultural resilience (Models 2 and 4). This is primarily attributed to the singular industrial structure in the central and northeastern regions, which are predominantly characterized as major grain-producing areas [45]. This characteristic results in agricultural resilience enhancement being more dependent on improvements in grain production efficiency and strengthened risk resistance capabilities. However, in Model 5, non-major grain-producing areas (0.669) demonstrate more efficient outcomes in rural industrial integration compared to major grain-producing areas (0.135). This result stems from major grain-producing areas being subject to stricter national food security policy constraints, which limit their industrial diversification potential. In contrast, non-major grain-producing areas possess more flexible resource allocation mechanisms and more comprehensive market-oriented industrial chains, facilitating more efficient industrial integration. This study not only echoes Tendall’s [105] multidimensional framework of agricultural resilience but also demonstrates the unique challenges faced by China as a major developing country in balancing food security and industrial development, extending beyond the applicability scope of developed country studies such as Darnhofer [106] Therefore, Hypothesis H4 is supported.

**Table 7:** Regional heterogeneity.

Variable Name	Model 1	Model 2	Model 3	Model 4	Model 5	Model 6
	Eastern Region	Central Region	Western Region	Northeastern Region	Major Grain-Producing Region	Non-Grain-Producing Region
*Rural Industrial Integration*	0.578*** (0.14)	0.266 (0.06)	0.477*** (0.12)	0.271 (0.19)	0.135* (0.08)	0.669*** (0.08)
*Digitalization Level*	0.056*** (0.01)	0.038* (0.02)	0.008* (0.01)	0.027 (0.03)	0.034*** (0.01)	0.012** (0.01)
*Transportation Network Density*	0.017 (0.03)	0.016 (0.01)	0.049** (0.02)	−0.497*** (0.12)	−0.041** (0.02)	0.055*** (0.02)
*Per Capita GDP*	0.192 (0.16)	0.147*** (0.05)	0.191** (0.08)	−0.081 (0.29)	0.256*** (0.05)	0.227*** (0.07)
*Robustness Test*	1.861** (0.91)	0.296*** (0.09)	0.513*** (0.1)	0.098 (0.85)	0.172** (0.09)	0.671*** (0.08)
*Individual Fixed Effects*	Yes	Yes	Yes	Yes	Yes	Yes
*Time Fixed Effects*	Yes	Yes	Yes	Yes	Yes	Yes
*Constant*	−3.612 (2.56)	−0.746*** (0.27)	−0.819** (0.43)	−1.574 (5.75)	−0.934*** (0.31)	−0.941** (0.39)
*R* ^*2*^	0.902	0.914	0.962	0.937	0.947	0.934
*DWS*	1.588	1.545	1.587	1.609	1.423	1.489

### 5.6 Robustness tests

This study employs three robustness testing methods: winsorization, exclusion of specific regions (municipalities directly under central government), and instrumental variable analysis (one-period lag). The results are presented in Table 8. First, the 1% winsorization results demonstrate (Model 1). The coefficient of rural industrial integration’s impact on agricultural resilience is 0.483, significant at the 1% level, which remains consistent with the baseline regression results. This confirms that the conclusion regarding rural industrial integration’s positive effect on agricultural resilience enhancement remains robust after eliminating the influence of outliers. Second, exclusion of municipalities directly under central government (Model 2). Considering that municipalities directly under central government differ substantially from ordinary provinces in terms of economic development levels and resource endowments, which may introduce estimation bias, this study excludes samples from Beijing, Shanghai, Tianjin, and Chongqing.The results indicate that the coefficient of rural industrial integration is 0.506, significant at the 1% level, and slightly higher than the baseline model. This further confirms that the positive impact of rural industrial integration on agricultural resilience remains valid even after excluding potential sample heterogeneity effects, and this effect may be more pronounced in non-municipality regions. Third, one-period lag analysis (Model 3). To address potential endogeneity issues, this study employs the one-period lagged level of rural industrial integration as an instrumental variable for analysis. The results show that after applying the instrumental variable method, the coefficient of rural industrial integration’s impact on agricultural resilience is 0.495, significant at the 1% level, which approximates the baseline model estimation results.

**Table 8:** Robustness tests.

Variable Name	Model 1	Model 2	Model 3
	1% Winsorization	Exclusion of Municipalities	One-Period Lag
*Rural Industrial Integration*	0.483*** (0.05)	0.506*** (0.07)	0.495*** (0.06)
*Digitalization Level*	0.027* (0.01)	0.031* (0.01)	0.025* (0.01)
*Transportation Network Density*	0.012*** (0.01)	0.015*** (0.01)	0.011** (0.01)
*Per Capita GDP*	0.213*** (0.04)	0.225*** (0.06)	0.209*** (0.05)
*Individual Fixed Effects*	Yes	Yes	Yes
*Time Fixed Effects*	Yes	Yes	Yes
*Constant*	−0.902*** (0.21)	−0.938*** (0.25)	−0.890*** (0.22)
*R* ^*2*^	0.936	0.951	0.937

### 5.7 Spatial spillover effects

Table 9 reports the global Moran’s I test results for rural industrial integration and agricultural resilience. The Moran’s I values for both rural industrial integration and agricultural resilience are positive and pass the significance test, providing preliminary verification that both variables exhibit spatial clustering characteristics.

**Table 9:** Spatial autocorrelation.

Year	Agriculture resilience			Rural industrial integration		
	Moran’s I	Z	P	Moran’s I	Z	P
2012	0.185	1.689	<0.89	0.268	1.735	0.086
2013	0.197	1.891	<0.79	0.269	1.772	0.069
2014	0.210	1.948	<0.05	0.278	1.865	0.062
2015	0.207	1.922	<0.05	0.253	1.741	0.087
2016	0.257	1.964	<0.05	0.278	2.128	0.062
2017	0.302	2.224	<0.05	0.252	1.975	<0.05
2018	0.335	2.833	<0.05	0.306	1.968	<0.05
2019	0.457	3.323	<0.01	0.398	2.844	<0.01
2020	0.448	3.299	<0.01	0.344	2.206	<0.05
2021	0.438	3.282	<0.01	0.374	2.407	<0.05
2022	0.510	3.802	<0.01	0.367	2.33	<0.05

Tables 10 and 11 demonstrates the positive spillover effects of the spatial Durbin model. First, this study validates the selection of the spatial Durbin model through Lagrange multiplier (LM) tests, where both the spatial error model (SEM) and spatial autoregressive model (SAR) yield p-values of 0.000 for both the standard and robust Lagrange multiplier tests. Second, the likelihood ratio (LR) test produces a p-value of 0.0000, indicating that the spatial Durbin model does not degenerate into either the spatial error model or the spatial autoregressive model. Furthermore, the Hausman test (comparing fixed effects and random effects) yields a chi-square statistic of 25.47 with 4 degrees of freedom and p < 0.001. Therefore, we reject the null hypothesis, confirming that the spatial Durbin model with time-fixed effects is the more appropriate specification. Additionally, the spatial lag coefficient of the dependent variable in the spatial weight matrix is 0.127, which is significant at the 1% level, indicating significant positive spatial spillover effects in agricultural resilience. It should be noted that the estimated coefficient ρ is significant and non-zero. Following LeSage [107], under these circumstances, partial differentiation should be employed to obtain unbiased estimates of the explanatory variables, requiring further decomposition into direct effects, indirect effects, and total effects.

**Table 10:** Spatial durbin model analysis.

Variable Name	Model 1	Model 2	Model 3	Model 4
*Rural Industrial Integration*	0.568*** (0.04)			
*Digitalization Level*		0.011** (0.01)		
*Transportation Network Density*			−0.043*** (0.01)	
*Per Capita GDP*				0.107** (0.05)
*W × Digitalization Level*	0.127*** (0.07)			
*W × Transportation Network Density*		−0.015*** (0.01)		
*W × Per Capita GDP*			0.022*** (0.06)	
*rho*				0.126** (0.06)
*Variance* *sigma2_e*	0.045*** (0.03)	0.045*** (0.03)	0.045*** (0.03)	0.045*** (0.03)
*Log-likelihood*	667.198			
*R* ^*2*^	0.948			

**Table 11:** Spatial spillover effects decomposition.

Variable Name	Direct Effect	Indirect Effect	Total Effect
*Rural Industrial Integration*	0.569*** (0.04)	0.131** (0.01)	0.700*** (0.06)
*Digitalization Level*	0.011*** (0.01)	−0.158*** (0.01)	−0.005 (0.01)
*Transportation Network Density*	−0.043*** (0.01)	0.022*** (0.01)	−0.207** (0.01)
*Per Capita GDP*	0.011** (0.05)	0.035 (0.06)	0.142*** (0.02)

Rural industrial integration significantly enhances agricultural resilience, demonstrated through direct positive effects on local agricultural resilience (0.569***) and spillover effects to neighboring regions through technology diffusion, resource sharing, and supply chain collaboration (0.131***). This indicates that the deep integration of primary, secondary, and tertiary industries in rural areas can optimize resource allocation and diversify farmers’ income sources, thereby strengthening agriculture’s risk resistance capacity and creating synergistic development effects across regions. However, while digitalization significantly improves local agricultural resilience (0.011***), the uneven distribution of digital resources across regions may exacerbate the digital divide and competitive effects, thereby inhibiting agricultural resilience enhancement in neighboring areas (−0.158***). Meanwhile, the expansion of transportation network density generates negative local effects (−0.043***), potentially due to land resource displacement and urbanization pressure caused by transportation infrastructure expansion, yet it produces positive spillover effects on agricultural resilience in neighboring regions through improved logistics efficiency and market connectivity (0.022***).

In summary, the spatial distribution and influencing mechanisms of agricultural resilience are shaped by multiple factors including industrial structure, technological levels, transportation networks, and economic development, which both demonstrate the potential for regional synergistic development and reveal challenges to agricultural resilience posed by urban-rural and regional development imbalances. Therefore, hypothesis H5 is confirmed.

## 6. Conclusions and recommendations

### 6.1 Research conclusions

As global climate change and market uncertainties pose severe challenges to economic and social systems, comprehensively enhancing the risk resistance capacity of agricultural systems has become a shared global responsibility. This study, based on the perspective of endogenous development dynamics of industrial integration, utilizes provincial panel data from 2012 to 2022 to construct a theoretical model of rural industrial integration and agricultural resilience, revealing the internal development mechanisms of agricultural resilience. The research findings indicate that, first, rural industrial integration significantly enhances agricultural resilience, with agricultural multifunctional expansion and improved benefit-linkage mechanisms being the most effective, followed by agricultural value chain extension and agri-service integration, while cultivation of new agricultural business models ranks last in effectiveness. Second, regarding the enhancement of agricultural resilience through rural industrial integration, the eastern and western regions demonstrate significant positive effects, while the central and northeastern regions, dominated by traditional grain production industries, fail to meet expectations, and the benefits of industrial integration in non-major grain-producing areas significantly exceed those in major grain-producing regions. Third, rural industrial integration can further elevate agricultural resilience levels through industrial structure rationalization, while industrial structure advancement and sophistication diminish the enhancing effects of rural industrial integration on agricultural resilience levels. Fourth, dynamic adjustments in urbanization levels attenuate the enhancing effects of rural industrial integration on agricultural resilience. Fifth, rural industrial integration not only enhances agricultural resilience in local regions but also generates spillover effects in neighboring areas.

### 6.2 Policy recommendations

First, implement differentiated pathways for industrial integration advancement. Agricultural sectors should proactively adapt to the new normal of economic development by supporting expansion toward multifunctional directions including ecological protection, cultural heritage preservation, and leisure experiences, utilizing policy support and financial guidance to achieve market-based realization of agricultural ecological, cultural, and social values. Innovation should focus on the “farmer + cooperative + enterprise” multi-party benefit-sharing model, establishing mechanisms for farmers to share value-added benefits across the entire industrial chain through diversified benefit distribution methods such as contract farming, equity cooperation, and guaranteed dividends, thereby enhancing farmers’ capacity to resist market risks. While steadily advancing industrial chain extension, priority support should be given to developing agricultural productive service industries, cultivating agricultural socialized service organizations that provide specialized services including comprehensive management, unified prevention and control, and drying and storage services to reduce agricultural production risks.

Second, implement regionally differentiated development strategies. Eastern regions should leverage their industrial foundations and market advantages to further deepen integration between agriculture and advanced manufacturing and high-end service industries, creating high-quality rural industrial integration demonstration zones. Western regions should capitalize on their ecological resource advantages by vigorously developing ecological agriculture, specialty agriculture, and eco-tourism, establishing new pathways for integrated development combining “ecology + agriculture + culture.” For central and northeastern major grain-producing regions, innovative industrial integration models should be developed, focusing on grain deep processing, premium grain brand development, and comprehensive grain industry chain service systems. Non-major grain-producing areas should be encouraged to develop specialty agricultural product processing, agricultural cultural and creative industries, and leisure agriculture, building regional specialty agricultural product brands.

Third, optimize pathways for industrial structure adjustment. Maintain industrial structure rationalization orientation, prioritizing optimization of internal agricultural structures and rational allocation between agriculture and related industries to ensure agricultural production stability and diversity. Cautious advancement is necessary to maintain appropriate scale and stability of basic agricultural industries, avoiding excessive displacement of agricultural resources by non-agricultural industries and ensuring food security and fundamental agricultural functions. Finally, industrial integration models should be innovated by exploring new pathways for technology-enabled agriculture such as digital agriculture and smart agriculture, promoting organic integration between traditional agriculture and modern technology.

Fourth, coordinate the relationship between urbanization and agricultural resilience. Establish urban-rural integrated development mechanisms by promoting bidirectional flow of urban-rural factors and improving rural property rights system reforms to revitalize rural land and capital resources. Cultivate new agricultural management entities by supporting development of family farms and farmer cooperatives, and guiding returning entrepreneurs to participate in modern agricultural development. Simultaneously, improve rural social security systems by establishing comprehensive pension and healthcare systems to reduce labor outflow caused by inadequate social protection and maintain reasonable rural population scales. Finally, innovate agricultural production methods by promoting intelligent and mechanized technologies to reduce labor dependence and improve productivity, effectively addressing labor shortages caused by urbanization.
